# Incidence of Nonkeratinocyte Skin Cancer After Breast Cancer Radiation Therapy

**DOI:** 10.1001/jamanetworkopen.2024.1632

**Published:** 2024-03-08

**Authors:** Shawheen J. Rezaei, Edward Eid, Jean Y. Tang, Allison W. Kurian, Bernice Y. Kwong, Eleni Linos

**Affiliations:** 1 Department of Dermatology, Stanford University, Stanford, California; 2 Center for Digital Health, Stanford University, Stanford, California; 3 Department of Medicine, Stanford University, Stanford, California; 4 Department of Epidemiology and Population Health, Stanford University, Stanford, California

## Abstract

**Importance:**

Previous studies have suggested that radiation therapy may contribute to an increased risk of subsequent nonkeratinocyte (ie, not squamous and basal cell) skin cancers.

**Objective:**

To test the hypothesis that radiation therapy for breast cancer increases the risk of subsequent nonkeratinocyte skin cancers, particularly when these cancers are localized to the skin of the breast or trunk.

**Design, Setting, and Participants:**

This population-based cohort study used longitudinal data from the Surveillance, Epidemiology, and End Results (SEER) Program for January 1, 2000, to December 31, 2019. The SEER database includes population-based cohort data from 17 registries. Patients with newly diagnosed breast cancer were identified and were evaluated for subsequent nonkeratinocyte skin cancer development. Data analysis was performed from January to August 2023.

**Exposures:**

Radiation therapy, chemotherapy, or surgery for breast cancer.

**Main Outcomes and Measures:**

The primary outcomes were standardized incidence ratios (SIRs) for subsequent nonkeratinocyte skin cancer development from 2000 to 2019 based on treatment type (radiation therapy, chemotherapy, or surgery), skin cancer site on the body, and skin cancer subtype.

**Results:**

Among the 875 880 patients with newly diagnosed breast cancer included in this study, 99.3% were women, 51.6% were aged older than 60 years, and 50.3% received radiation therapy. A total of 11.2% patients identified as Hispanic, 10.1% identified as non-Hispanic Black, and 69.5% identified as non-Hispanic White. From 2000 to 2019, there were 3839 patients with nonkeratinocyte skin cancer, including melanoma (3419 [89.1%]), Merkel cell carcinoma (121 [3.2%]), hemangiosarcoma (104 [2.7%]), and 32 other nonkeratinocyte skin cancers (195 [5.1%]), documented to occur after breast cancer treatment. The risk of nonkeratinocyte skin cancer diagnosis after breast cancer treatment with radiation was 57% higher (SIR, 1.57 [95% CI, 1.45-1.7]) than that of the general population when considering the most relevant site: the skin of the breast or trunk. When risk at this site was stratified by skin cancer subtype, the SIRs for melanoma and hemangiosarcoma were both statistically significant at 1.37 (95% CI, 1.25-1.49) and 27.11 (95% CI, 21.6-33.61), respectively. Receipt of radiation therapy was associated with a greater risk of nonkeratinocyte skin cancer compared with chemotherapy and surgical interventions.

**Conclusions and Relevance:**

In this study of patients with breast cancer, an increased risk of melanoma and hemangiosarcoma after breast cancer treatment with radiation therapy was observed. Although occurrences of nonkeratinocyte skin cancers are rare, physicians should be aware of this elevated risk to help inform follow-up care.

## Key Points

**Question:**

Are patients with breast cancer who receive radiation therapy at a higher risk of developing subsequent nonkeratinocyte (ie, not squamous and basal cell) skin cancers?

**Findings:**

This cohort study of 875 880 patients with newly diagnosed breast cancer found a significantly increased risk of development of posttreatment nonkeratinocyte skin cancers, particularly melanoma and hemangiosarcoma.

**Meaning:**

These results suggest that although nonkeratinocyte skin cancer is a rare occurrence, physicians caring for patients with breast cancer should be aware of this elevated risk to help inform follow-up care for survivors.

## Teaser

This cohort study examines the association between radiation therapy for breast cancer and increased risk of subsequent nonkeratinocyte skin cancer development.

## Introduction

Overall incidence rates of cancer have declined in the US over the past decade, yet cancer remains the second leading cause of death.^1,2^ As of 2022, there are more than 18 million cancer survivors in the US, with more than 4 million survivors of breast cancer, the most prevalent cancer type among women.^3^ Although improved treatments have resulted in relatively high cure rates of early-stage breast cancer,^4^ survivors nevertheless experience numerous challenges after treatment, including burdens to their physical and mental health.^5^ There is also a risk of recurrence from the breast cancer metastasizing to another part of the body or increased susceptibility to a second primary cancer in a new location.^6,7^ Although subsequent cancers may arise due to chance, there is well-established evidence that certain types of breast cancer treatments, such as radiation therapy, result in an increased risk of secondary cancer and damage to surrounding tissues.^8,9,10^

The role of radiation therapy in subsequent cancer development has been studied for a range of primary cancers, including those of the prostate and rectum,^11^ head and neck,^12^ and breast.^13^ Much of the literature focuses on secondary lung cancer or cardiovascular disease risk after breast cancer radiation therapy.^13,14^ Yet less is known about the long-term overall risk of subsequent nonkeratinocyte skin cancers after breast cancer radiation therapy or other treatments. In a study published in 2004 using data from 1973 to 1999, researchers found a positive association between radiation therapy for breast cancer and cutaneous melanoma.^15^ A study of malignant neoplasms after breast cancer treatment with radiation and surgery from 1968 to 1987 reported an increased risk of melanoma, although the sample size was small. Therefore, while an association between breast cancer treatment and skin cancer remains possible, a large population-based study of more recent data may better characterize this risk for melanoma and for nonkeratinocyte skin cancers, which are understudied.

An improved understanding of the risk profiles for various skin cancers after breast cancer treatments can have important implications for monitoring and follow-up care. This study leverages the National Cancer Institute (NCI) Survey, Epidemiology, and End Results (SEER) Program database to analyze the risk of subsequent nonkeratinocyte (ie, not squamous or basal cell) skin cancers after breast cancer treatment. In addition to assessment of overall risk, we sought to identify disaggregated risks based on treatment modalities, subsequent cancer sites, and skin cancer subtypes. We hypothesized that the incidence of nonkeratinocyte skin cancers increases when radiation therapy is administered, both when given alone and in conjunction with other treatment modalities. We also hypothesized that the magnitude of this increase is greater when subsequent nonkeratinocyte skin cancers are confined to the skin of the breast or trunk, because this is the area most likely to be the direct target of radiation.

## Methods

This cohort study was exempt from review by the Stanford University Institutional Review Board due to the use of only deidentified data, and the need for informed consent was waived. The study followed the Strengthening the Reporting of Observational Studies in Epidemiology (STROBE) reporting guideline.

### Data Source

The study used population-based NCI SEER data for the analysis. Data from 17 registries spanning from January 1, 2000, to December 31, 2019, were included.

### Statistical Analysis

We used SEER*Stat software, version 8.4.1 (NCI Surveillance Research Program), to perform the multiple primary-standardized incidence ratio (MP-SIR) session analysis in June 2023. The MP-SIR session enables the linkage of a primary cancer exposure to subsequent cancer events for individual records. When these are assessed in aggregate, an SIR can be assessed for the subsequent event, calculated as the incidence rate of an event observed in the population of interest divided by the incidence rate expected in a standard population. The SIRs are adjusted by sex, race and ethnicity, age, and year of initial diagnosis (as identified by the SEER database), and they can be used as a statistical measure of risk for subsequent cancer diagnosis and have associated CIs and significance levels. Data on race (American Indian or Alaska Native, Asian or Pacific Islander, Black, White, or unknown) and ethnicity (Hispanic) are reported as identified in the SEER database and were collected primarily for the adjustment of SIRs.

For the purposes of this study, SIRs were calculated to assess the risk of subsequent nonkeratinocyte skin cancers after a primary breast cancer diagnosis, stratified by various breast cancer treatment modalities. The main treatment modality examined was radiation therapy for breast cancer, and chemotherapy and surgical intervention for breast cancer were evaluated in combination with radiation therapy. An initial breast cancer diagnosis and a subsequent nonkeratinocyte skin cancer diagnosis were determined using the *International Classification of Diseases for Oncology, Third Edition* (*ICD-O-3*) Site Recode/World Health Organization 2008 definition.^16^ The SEER Program does not collect data on squamous or basal cell carcinomas of the skin, and subsequent nonkeratinocyte skin cancer diagnoses were thus assessed for melanoma, hemangiosarcoma (synonymous with angiosarcoma in the SEER database), Merkel cell carcinoma, and other rare nonkeratinocyte skin cancers. The year of diagnosis for breast cancer was limited to 2000 to 2018, whereas the year of diagnosis for nonkeratinocyte skin cancer was extended for an additional year (2000-2019) to allow for greater lead time between primary and subsequent cancer diagnoses. Similarly, the default SEER*Stat latency exclusion period of 2 months was applied to avoid counting nonkeratinocyte skin cancers occurring immediately after a breast cancer diagnosis.

*P* < .05 (2-tailed) was deemed statistically significant. Data analysis was performed from January to August 2023.

## Results

Among the 875 880 patients with newly diagnosed breast cancer included in this study, 99.3% were women and 0.7% were men (Table 1). Half of the patients were aged older than 60 years (51.6%) and had received radiation therapy (50.3%). In terms of race and ethnicity, 0.4% of patients identified as American Indian or Alaska Native, 8.4% as Asian or Pacific Islander, 10.1% as Black, 11.2% as Hispanic, 69.5% as White, and 0.5% as being of non-Hispanic unknown race. With regard to treatment, 41.2% of patients with breast cancer received chemotherapy, and the most common surgical interventions were partial mastectomy (52.7%), modified radical mastectomy (19.5%), and total mastectomy (17.8%). Half of the patients (52.1%) were from the upper 2 income quintiles, and the majority (86.1%) resided in urban areas.

**Table 1.** Characteristics of Patients With Breast Cancer by Radiation Therapy Receipt, 2000 to 2018^a^

Characteristic^b^	Total (N = 875 880)	Radiation therapy received	
		No or unknown (n = 435 049)	Yes (n = 440 831)
Sex			
Male	6112 (0.7)	4465 (1.0)	1647 (0.4)
Female	869 768 (99.3)	430 584 (99.0)	439 184 (99.6)
Race and ethnicity			
American Indian or Alaska Native	3823 (0.4)	1909 (0.4)	1914 (0.4)
Asian or Pacific Islander	73 377 (8.4)	37 461 (8.6)	35 916 (8.2)
Black	87 988 (10.1)	46 192 (10.6)	41 796 (9.5)
Hispanic	98 211 (11.2)	51 836 (11.9)	46 375 (10.5)
White	608 363 (69.5)	294 780 (67.8)	313 583 (71.1)
Unknown	4118 (0.5)	2871 (0.7)	1247 (0.3)
Age range, y			
0-19	91 (0.0)	66 (0.0)	25 (0.0)
20-39	49 829 (5.7)	25 856 (5.9)	23 973 (5.4)
40-59	373 756 (42.7)	173 873 (40.0)	199 883 (45.3)
60-79	367 251 (41.9)	172 904 (39.7)	194 347 (44.1)
≥80	84 953 (9.7)	62 350 (14.3)	22 603 (5.1)
Chemotherapy administered			
No or not known	514 772 (58.8)	280 103 (64.4)	234 669 (53.2)
Yes	361 108 (41.2)	154 946 (35.6)	206 162 (46.8)
Surgical intervention			
Partial mastectomy^c^	461 175 (52.7)	121 637 (28.0)	339 538 (77.0)
Subcutaneous mastectomy	9052 (1.0)	6880 (1.6)	2172 (0.5)
Total mastectomy	156 281 (17.8)	126 930 (29.2)	29 351 (6.7)
Modified radical mastectomy	171 129 (19.5)	114 779 (26.4)	56 350 (12.8)
None	68 200 (7.8)	57 254 (13.2)	10 946 (2.5)
Local tumor destruction, NOS	96 (0.0)	76 (0.0)	20 (<0.01)
Bilateral mastectomy for single tumor involving both breasts	89 (0.0)	58 (0.0)	31 (0.0)
Radical mastectomy	2904 (0.3)	1811 (0.4)	1093 (0.3)
Extended radical mastectomy	170 (0.0)	97 (0.0)	73 (0.0)
Mastectomy, NOS	2800 (0.3)	2057 (0.5)	743 (0.2)
Surgery, NOS	1240 (0.1)	861 (0.2)	379 (0.1)
Unknown	2744 (0.3)	2609 (0.6)	135 (0.0)
SES quintile	817 807	386 533	431 274
1 (Lowest)	100 973 (12.3)	43 307 (11.2)	57 666 (13.4)
2	114 889 (14.0)	51 413 (13.3)	63 476 (14.7)
3	135 709 (16.6)	64 081 (16.6)	71 628 (16.6)
4	178 322 (21.8)	86 633 (22.4)	91 689 (21.3)
5 (Highest)	247 680 (30.3)	123 306 (31.9)	124 374 (28.8)
Unknown	40 234 (4.9)	17 793 (4.6)	22 441 (5.2)
Rural vs urban residence^c^			
All urban	548 574 (67.1)	257 994 (66.7)	2 902 580 (67.4)
Mostly urban	155 905 (19.1)	75 971 (19.7)	79 934 (18.5)
Mostly rural	46 810 (5.7)	22 653 (5.9)	24 157 (5.6)
All rural	37 985 (4.6)	17 801 (4.6)	20 184 (4.7)
Unknown	28 533 (3.5)	12 114 (3.1)	16 419 (3.8)

Abbreviations: NOS, not otherwise specified; SES, socioeconomic status.

^a^ Values are presented as No. (%) of patients.

^b^ As identified by the Surveillance, Epidemiology, and End Results Program database.

^c^ Data were limited to 2006 to 2018.

There were 3839 patients with nonkeratinocyte skin cancer, including melanoma (3419 [89.1%]), Merkel cell carcinoma (121 [3.2%]), hemangiosarcoma (104 [2.7%]), and 32 other nonkeratinocyte skin cancers (195 [5.1%]), after breast cancer treatment. Table 2 presents the SIRs for skin cancer after primary breast cancer diagnosis, stratified by radiation therapy status and skin cancer location on the body (ie, all skin vs skin of the breast or trunk). Across all parts of the body, the incidence rate for subsequent nonkeratinocyte skin cancer after breast cancer diagnosis was 26% higher than in the general population if radiation therapy was given (SIR, 1.26 [95% CI, 1.21-1.32]) and 8% higher than in the general population with no or unknown radiation therapy receipt (SIR, 1.08 [95% CI, 1.03-1.13]).

**Table 2.** SIRs for Subsequent Nonkeratinocyte Skin Cancer After Breast Cancer Treatment, Stratified by Radiation Therapy Receipt and Subsequent Skin Cancer Site, 2000 to 2019

Subsequent skin cancer site and type	Incidence rate^a^		SIR (95% CI)^b^	Excess risk per 100 000 persons/y
	Observed	Expected		
**All skin**				
No radiation received or not known				
Total	1657	1535.73	1.08 (1.03-1.13)^c^	3.8
Melanoma	1495	1396.01	1.07 (1.02-1.13)^c^	3.1
Hemangiosarcoma	17	5.89	2.89 (1.68-4.62)^c^	0.4
Merkel cell carcinoma	62	52.57	1.18 (0.90-1.51)	0.3
Other nonepithelial	83	81.26	1.02 (0.81-1.27)	0.1
Radiation received				
Total	2182	1727.83	1.26 (1.21-1.32)^c^	12.8
Melanoma	1924	1584.97	1.21 (1.16-1.27)^c^	9.6
Hemangiosarcoma	87	5.95	14.63 (11.72-18.04)^c^	2.3
Merkel cell carcinoma	59	52.1	1.13 (0.86-1.46)	0.2
Other nonepithelial	112	84.8	1.32 (1.09-1.59)^c^	0.8
**Breast or trunk**				
No radiation received or not known				
Total	379	339.74	1.12 (1.01-1.23)^c^	1.2
Melanoma	345	313.82	1.10 (0.99-1.22)	1
Hemangiosarcoma	11	2.84	3.87 (1.93-6.93)^c^	0.3
Merkel cell carcinoma	6	3.96	1.52 (0.56-3.30)	0.1
Other nonepithelial	17	19.13	0.89 (0.52-1.42)	−0.1
Radiation received				
Total	616	393.1	1.57 (1.45-1.70)^c^	6.3
Melanoma	499	365.38	1.37 (1.25-1.49)^c^	3.8
Hemangiosarcoma	83	3.06	27.11 (21.6-33.61)^c^	2.3
Merkel cell carcinoma	5	3.96	1.26 (0.41-2.95)	0
Other nonepithelial	29	20.7	1.40 (0.94-2.01)	0.2

Abbreviation: SIR, standardized incidence ratio.

^a^ To determine the SIR, the incidence rate of an event observed in the population of interest was divided by the incidence rate expected in a standard population.

^b^ Because the SEER*Stat software does not provide exact *P* values in the multiple primary SIR session, values are reported as statistically significant at *P* < .05.

^c^ *P* < .05.

The risks for specific nonkeratinocyte skin cancer subtypes were also assessed. For melanoma, the SIR was 1.21 (95% CI, 1.16-1.27) for radiation therapy receipt and 1.07 (95% CI, 1.02-1.13) for no or unknown radiation therapy receipt for breast cancer. The SIR of subsequent hemangiosarcoma was 14.63 (95% CI, 11.72-18.04) for radiation therapy receipt and 2.89 (95% CI, 1.68-4.62) for no or unknown radiation therapy receipt. The SIRs for Merkel cell carcinoma were not statistically significant. A total of 32 other nonkeratinocyte skin cancer subtypes were also documented to occur after breast cancer treatment (Table 3). The most common other nonkeratinocyte skin cancers by *ICD-O-3* code were as follows: sebaceous adenocarcinoma (54 [27.7%]); dermatofibrosarcoma, not otherwise specified (28 [14.4%]); malignant eccrine poroma (14 [7.2%]); skin appendage carcinoma (14 [7.2%]); malignant fibrous histiocytoma (14 [7.2%]); and sclerosing sweat duct carcinoma (13 [6.7%]). The SIRs for these other nonkeratinocyte skin cancers after breast cancer treatment were 1.32 (95% CI, 1.09-1.59) for radiation therapy receipt and 1.02 (95% CI, 0.81-1.27) for no or unknown radiation therapy receipt for breast cancer.

**Table 3.** Other Nonkeratinocyte Skin Cancers Developed After Primary Breast Cancer, Sorted by Frequency, 2000 to 2019

*ICD-O-3* code	Radiation therapy received for primary breast cancer, No. of patients		Total No. (%) of patients
	No or unknown	Yes	
8410/3: Sebaceous adenocarcinoma	25	29	54 (27.7)
8832/3: Dermatofibrosarcoma, NOS	12	16	28 (14.4)
8409/3: Eccrine poroma, malignant	7	7	14 (7.2)
8390/3: Skin appendage carcinoma	7	7	14 (7.2)
8830/3: Malignant fibrous histiocytoma	4	10	14 (7.2)
8407/3: Sclerosing sweat duct carcinoma	4	9	13 (6.7)
8413/3: Eccrine adenocarcinoma	3	6	9 (4.6)
8140/3: Adenocarcinoma, NOS	3	2	5 (2.6)
8200/3: Adenoid cystic carcinoma	1	4	5 (2.6)
8802/3: Giant cell sarcoma	2	3	5 (2.6)
8480/3: Mucinous adenocarcinoma	3	1	4 (2.1)
8542/3: Paget disease, extramammary (except Paget disease of bone)	1	3	4 (2.1)
8401/3: Apocrine adenocarcinoma	1	2	3 (1.5)
8403/3: Malignant eccrine spiradenoma	1	1	2 (1.0)
8400/3: Sweat gland adenocarcinoma	0	2	2 (1.0)
8402/3: Nodular hidradenoma, malignant	1	1	2 (1.0)
8560/3: Adenosquamous carcinoma	1	1	2 (1.0)
8710/3: Glomangiosarcoma	0	1	1 (0.5)
8201/3: Cribriform carcinoma, NOS	0	1	1 (0.5)
8143/3: Superficial spreading adenocarcinoma	1	0	1 (0.5)
8540/3: Paget disease, mammary	1	0	1 (0.5)
8260/3: Papillary adenocarcinoma, NOS	0	1	1 (0.5)
8940/3: Mixed tumor, malignant, NOS	1	0	1 (0.5)
9170/3: Lymphangiosarcoma	0	1	1 (0.5)
8408/3: Eccrine papillary adenocarcinoma	0	1	1 (0.5)
9580/3: Granular cell tumor, malignant	0	1	1 (0.5)
8833/3: Pigmented dermatofibrosarcoma protuberans	0	1	1 (0.5)
8520/3: Lobular carcinoma, NOS	1	0	1 (0.5)
8850/3: Liposarcoma, NOS	0	1	1 (0.5)
8890/3: Leiomyosarcoma, NOS	0	1	1 (0.5)
8804/3: Epithelioid sarcoma	0	1	1 (0.5)
8711/3: Glomus tumor, malignant	0	1	1 (0.5)
Total	80	115	195 (100)

Abbreviations: *ICD-O-3*, *International Classification of Diseases for Oncology, Third Edition*; NOS, not otherwise specified.

Because the effects of radiation therapy on subsequent nonkeratinocyte skin cancer pathology were hypothesized to be greater at the site of radiation administration, SIRs were also calculated for skin cancers localized to the breast or trunk area. Across all skin cancers studied, the SIRs were greater for skin cancers localized to the breast or trunk compared with all skin sites combined (Table 2). When localized to skin of the breast or trunk, there was a 1.37-fold (SIR, 1.37 [95% CI, 1.25-1.49]) increase in the incidence of melanoma and a 27.11-fold (SIR, 27.11 [95% CI, 21.6-33.61]) increase in the incidence of hemangiosarcoma in this area after radiation compared with the general population. The incidence rate of all nonkeratinocyte skin cancers combined in this area after breast cancer treatment was 57% higher than in the general population if radiation therapy was given (SIR, 1.57 [95% CI, 1.45-1.7]) and 12% higher than in the general population with no or unknown radiation therapy (SIR, 1.12 [95% CI, 1.01-1.23]).

The use of other breast cancer treatment modalities was also explored. Of the 995 patients with breast cancer that preceded a subsequent nonkeratinocyte skin cancer in the breast or trunk area, 616 (61.9%) were treated with radiation therapy, 399 (40.1%) with chemotherapy, and 975 (98.0%) with some form of surgical intervention. The SIRs for skin cancers in the breast or trunk area by chemotherapy and radiation therapy for breast cancer are provided in Table 4. When both radiation and chemotherapy were not given or uncertain, the incidence of skin cancers in this area was not significantly different from that of the general population (SIR, 1.04 [95% CI, 0.91-1.19]). When chemotherapy was given, there was a statistically significant 25% (SIR, 1.25 [95% CI, 1.06-1.47]) and 46% (SIR, 1.46 [95% CI, 1.28-1.65]) higher incidence of subsequent nonkeratinocyte skin cancers with no or unknown radiation therapy and radiation therapy, respectively. When radiation therapy was given in the absence of chemotherapy treatment, the SIR was highest (SIR, 1.65 [95% CI, 1.49-1.83]). There was no statistically significant increase in the SIR for subsequent nonkeratinocyte skin cancers for surgical interventions when radiation therapy was not given (Table 5).

**Table 4.** SIRs for Subsequent Nonkeratinocyte Skin Cancer of the Breast or Trunk After Breast Cancer Treatment, Stratified by Radiation Therapy and Chemotherapy, 2000 to 2019

Treatment type	Incidence rate^a^		SIR (95% CI)^b^	Excess risk per 100 000 persons/y
	Observed	Expected		
None	231	221.62	1.04 (0.91-1.19)	0.5
Chemotherapy alone	148	118.12	1.25 (1.06-1.47)^c^	2.6
Radiation therapy alone	365	220.93	1.65 (1.49-1.83)^c^	7.7
Both radiation and chemotherapy	251	172.17	1.46 (1.28-1.65)^c^	4.7

Abbreviation: SIR, standardized incidence ratio.

^a^ To determine the SIR, the incidence rate of an event observed in the population of interest was divided by the incidence rate expected in a standard population.

^b^ Because the SEER*Stat software does not provide exact *P* values in the multiple primary SIR session, values are reported as statistically significant at *P* < .05.

^c^ *P* < .05.

**Table 5.** SIRs for Subsequent Nonkeratinocyte Skin Cancer of the Breast or Trunk After Breast Cancer Treatment, Stratified by Radiation Therapy and Common Surgeries (2000-2019)

Radiation therapy and surgical intervention type	Incidence rate^a^		SIR (95% CI)^b^	Excess risk per 100 000 persons/y
	Observed	Expected		
No radiation received or not known				
Partial mastectomy^c^	110	101.43	1.08 (0.89-1.31)	0.9
Total mastectomy	111	99.42	1.12 (0.92-1.34)	1.3
Modified radical mastectomy	131	109.74	1.19 (1-1.42)	2
None	14	21.3	0.66 (0.36-1.1)	−3.5
Radiation received				
Partial mastectomy^c^	498	323.75	1.54 (1.41-1.68)^d^	6.1
Total mastectomy	37	19.02	1.95 (1.37-2.68)^d^	10
Modified radical mastectomy	73	43.81	1.67 (1.31-2.09)^d^	6.8
None	4	4.02	0.99 (0.27-2.55)	−0.1

Abbreviation: SIR, standardized incidence ratio.

^a^ The SIR was calculated as the incidence rate of an event observed in the population of interest divided by the incidence rate expected in a standard population.

^b^ Because the SEER*Stat software does not provide exact *P* values in the multiple primary SIR session, values are reported as statistically significant at *P* < .05.

^c^ Includes lumpectomy.

^d^ *P* < .05.

The SIRs of subsequent nonkeratinocyte skin cancers in the breast or trunk area were also disaggregated based on race defined as Black, White, and other races (American Indian or Alaska Native, or Asian or Pacific Islander) by the SEER database. With no or unknown radiation therapy, only White patients with breast cancer had a statistically significant increase in the incidence of subsequent nonkeratinocyte skin cancers (SIR, 1.19 [95% CI, 1.07-1.31]); however, there was a statistically significant increase in nonkeratinocyte skin cancer incidence across race when radiation therapy was given. Most notably, Black individuals and individuals of other races had 4.29-fold (SIR, 4.29 [95% CI, 2.28-7.33]) and 3.53-fold (SIR, 3.53 [95% CI, 1.69-6.5]) increases in subsequent nonkeratinocyte skin cancer incidence, respectively.

## Discussion

This study of 875 880 patients with breast cancer investigated the incidence of subsequent nonkeratinocyte skin cancer diagnoses, particularly after administration of radiation therapy and other treatment modalities. We observed a statistically significant increased risk of nonkeratinocyte skin cancer development after breast cancer treatment. The risk of subsequent nonkeratinocyte skin cancers was further elevated when radiation therapy was given. Thirty-five different subtypes of nonkeratinocyte skin cancer occurred after breast cancer treatment. Among these, melanoma, hemangiosarcoma, and Merkel cell carcinoma were the most common. Both melanoma and hemangiosarcoma had statistically significant elevated risks of development after breast cancer radiation, especially on the breast or trunk. Notably, hemangiosarcoma had the highest (27.11-fold) increase. These findings point to the important role of radiation therapy in the development of subsequent nonkeratinocyte skin cancers.

Skin cancers have multifactorial causes, including genetic predisposition and environmental risk factors.^17,18^ Among environmental risk factors, UV radiation exposure has been widely characterized as influencing skin cancer development.^18,19^ In light of the modifiable nature of this risk factor, previous studies have emphasized prevention strategies, such as using sunscreen and other forms of sun protection and avoiding indoor tanning.^20,21^ Cancer treatment, however, also carries a risk of subsequent nonkeratinocyte skin cancer development that is more difficult to modify. For instance, radiation therapy is an integral treatment modality for cancer, but it is known to cause adverse side effects, including potential toxic effects in the skin.^22,23,24^ In a study of childhood cancer survivors diagnosed in 1970 to 1986, researchers found an elevated risk of melanoma in adulthood.^25^ Our study corroborates the finding of a 46% elevated risk in cutaneous melanoma after radiation therapy for breast cancer in a 2004 study.^15^ Other cancer treatment modalities may also elevate the risk of subsequent nonkeratinocyte skin cancers when combined with radiation therapy. For example, although rare, hemangiosarcoma and lymphedema have been known to develop after radiation therapy and radical mastectomy, respectively, which our findings corroborate.^23,26^

The findings of this study are consistent with the well-established role of radiation therapy as a risk factor for secondary malignant neoplasms. Cancer treatment modalities each have their own risk-benefit profile that includes potential adverse reactions and other side effects. Given that radiation therapy results in a notable reduction of both breast cancer recurrence and mortality,^27^ the overall small rate of subsequent nonkeratinocyte skin cancer (<1% for all patients with breast cancer and for those treated with radiation therapy) does not necessarily merit altering treatment decision making or surveillance recommendations. Serious skin cancers such as melanoma can be effectively treated when caught early, yet they can metastasize with fatal consequences if left unmonitored and untreated. There is mixed evidence, however, on the benefits of patient self-surveillance through skin checks. A study of childhood cancer survivors treated with radiation found that 13% of survivors had conducted a skin self-examination in the past 2 months and had received a physician whole-body examination in the past year.^28^ Although a cohort study found that melanomas identified through routine skin checks were associated with reduced all-cause mortality, skin checks were not found to reduce melanoma-specific mortality.^29^ Nevertheless, our findings may help inform physicians that their patients with breast cancer are at an elevated but small risk of subsequent nonkeratinocyte skin malignant neoplasms after radiation therapy. There is a need to better define and incorporate subsequent risk of malignant neoplasms within patient consultation processes and survivorship follow-up care plans.

### Limitations

There are several limitations to this study. The SEER program does not provide a comprehensive family history or genetic profiles for patients included in the database. Thus, it is not possible to stratify the incidence of subsequent nonkeratinocyte skin cancers based on germline pathogenic variants that may predispose an individual to a greater risk of developing melanoma or other skin cancers. The SEER program also excludes squamous cell carcinoma and basal cell carcinoma, the 2 most common skin cancers. Although these 2 skin cancers carry a much lower mortality risk than melanoma, they nevertheless have high incidence rates that merit further investigation.^30^ Future studies could explore the risk of these skin cancer subtypes after breast cancer treatment. Additionally, there are limitations in how the SEER database reports radiation therapy (ie, no and unknown radiation therapy administration are combined) and previous studies have shown underascertainment of radiation therapy in the SEER data.^31^ As such, the incidence of skin cancer may be even lower in individuals who have definitively not received radiation compared with the none or unknown category displayed in the analysis. No reported time of follow-up was included in this study. If patients had a very short median follow-up time, for example, the risk for subsequent cancer may have been underestimated, as malignant neoplasms that take much longer to develop may not be reflected. Even when limiting the site of nonkeratinocyte skin cancers to the breast or trunk, this study was not able to ascertain the precise location of radiation therapy, which would better reflect the site at risk of subsequent cancer development.

## Conclusions

This cohort study is the first, to our knowledge, to characterize the risk of nonkeratinocyte skin cancer subtypes after breast cancer radiation therapy. We observed an overall increased risk of subsequent nonkeratinocyte skin cancers, specifically for melanoma and hemangiosarcoma, after breast cancer radiation therapy. These findings suggest that when the skin cancer site is localized to the skin of the breast or trunk, the magnitude of this risk increases. Further investigations could explore the effects of radiation dosage and genetic profiles of patients with breast cancer as potential contributors to this elevated risk.

## Notes

Supplement.
Data Sharing Statement

## References

[1] Centers for Disease Control and Prevention. U.S. cancer statistics: highlights from 2020 mortality and incidence with comparison to 2019 incidence to assess the effect of the COVID-19 pandemic. U.S. Cancer Statistics Data Briefs, No. 35. June 2023. Accessed February 6, 2024. https://www.cdc.gov/cancer/uscs/about/data-briefs/no35-USCS-highlights-2020.htm

[2] Siegel RL, Miller KD, Wagle NS, Jemal A. Cancer statistics, 2023. CA Cancer J Clin. 2023;73(1):17-48. doi:10.3322/caac.21763 36633525

[3] Miller KD, Nogueira L, Devasia T, . Cancer treatment and survivorship statistics, 2022. CA Cancer J Clin. 2022;72(5):409-436. doi:10.3322/caac.21731 35736631

[4] Hölzel D, Eckel R, Bauerfeind I, . Improved systemic treatment for early breast cancer improves cure rates, modifies metastatic pattern and shortens post-metastatic survival: 35-year results from the Munich Cancer Registry. J Cancer Res Clin Oncol. 2017;143(9):1701-1712. doi:10.1007/s00432-017-2428-0 28429102 PMC11819226

[5] Harbeck N, Penault-Llorca F, Cortes J, . Breast cancer. Nat Rev Dis Primers. 2019;5(1):66. doi:10.1038/s41572-019-0111-2 31548545

[6] Marino N, Woditschka S, Reed LT, . Breast cancer metastasis: issues for the personalization of its prevention and treatment. Am J Pathol. 2013;183(4):1084-1095. doi:10.1016/j.ajpath.2013.06.012 23895915 PMC3791679

[7] Early AP, Moon W. Breast cancer and secondary cancer recurrences after autologous tissue reconstruction. Clin Breast Cancer. 2021;21(1):e96-e101. doi:10.1016/j.clbc.2020.07.015 32855081

[8] Vogel M, Gade J, Timm B, . Comparison of breast cancer radiotherapy techniques regarding secondary cancer risk and normal tissue complication probability—modelling and measurements using a 3D-printed phantom. Front Oncol. 2022;12:892923. doi:10.3389/fonc.2022.892923 35965556 PMC9365503

[9] Zhang Q, Liu J, Ao N, . Secondary cancer risk after radiation therapy for breast cancer with different radiotherapy techniques. Sci Rep. 2020;10(1):1220. doi:10.1038/s41598-020-58134-z 31988348 PMC6985127

[10] Paganetti H, Depauw N, Johnson A, Forman RB, Lau J, Jimenez R. The risk for developing a secondary cancer after breast radiation therapy: comparison of photon and proton techniques. Radiother Oncol. 2020;149:212-218. doi:10.1016/j.radonc.2020.05.035 32464163 PMC11293368

[11] Lee YC, Hsieh CC, Li CY, Chuang JP, Lee JC. Secondary cancers after radiation therapy for primary prostate or rectal cancer. World J Surg. 2016;40(4):895-905. doi:10.1007/s00268-015-3324-x 26711638

[12] Brook I. Late side effects of radiation treatment for head and neck cancer. Radiat Oncol J. 2020;38(2):84-92. doi:10.3857/roj.2020.00213 33012151 PMC7533405

[13] Figlia V, Simonetto C, Eidemüller M, . Mammary chain irradiation in left-sided breast cancer: can we reduce the risk of secondary cancer and ischaemic heart disease with modern intensity-modulated radiotherapy techniques? Breast Care (Basel). 2021;16(4):358-367. doi:10.1159/000509779 34602941 PMC8436658

[14] Pignol JP, Hoekstra N, Wilke D, Dahn H, Nolan M, Vicini F. Estimation of annual secondary lung cancer deaths using various adjuvant breast radiotherapy techniques for early-stage cancers. Front Oncol. 2021;11:713328. doi:10.3389/fonc.2021.713328 34434899 PMC8381359

[15] Goggins W, Gao W, Tsao H. Association between female breast cancer and cutaneous melanoma. Int J Cancer. 2004;111(5):792-794. doi:10.1002/ijc.20322 15252852

[16] National Cancer Institute. Site Recode *ICD-O-3*/WHO 2008 definition. Surveillance, Epidemiology, and End Results Program. Accessed June 29, 2023. https://seer.cancer.gov/siterecode/icdo3_dwhoheme/index.html

[17] Laskar R, Ferreiro-Iglesias A, Bishop DT, ; Australian Melanoma Family Study Investigators; Leeds Case-Control Study Investigators. Risk factors for melanoma by anatomical site: an evaluation of aetiological heterogeneity. Br J Dermatol. 2021;184(6):1085-1093. doi:10.1111/bjd.19705 33270213 PMC9969114

[18] Carr S, Smith C, Wernberg J. Epidemiology and risk factors of melanoma. Surg Clin North Am. 2020;100(1):1-12. doi:10.1016/j.suc.2019.09.005 31753105

[19] Perez M, Abisaad JA, Rojas KD, Marchetti MA, Jaimes N. Skin cancer: primary, secondary, and tertiary prevention. Part I. J Am Acad Dermatol. 2022;87(2):255-268. doi:10.1016/j.jaad.2021.12.066 35176397

[20] Linos E, Katz KA, Colditz GA. Skin cancer—the importance of prevention. JAMA Intern Med. 2016;176(10):1435-1436. doi:10.1001/jamainternmed.2016.5008 27459394 PMC5489348

[21] Jin J. Screening and prevention of skin cancer. JAMA. 2023;329(15):1324. doi:10.1001/jama.2023.4045 37071093

[22] Fitzgerald TJ, Jodoin MB, Tillman G, . Radiation therapy toxicity to the skin. Dermatol Clin. 2008;26(1):161-172, ix. doi:10.1016/j.det.2007.08.005 18023776

[23] Bonito FJP, de Almeida Cerejeira D, Dahlstedt-Ferreira C, Oliveira Coelho H, Rosas R. Radiation-induced angiosarcoma of the breast: a review. Breast J. 2020;26(3):458-463. doi:10.1111/tbj.13504 31448482

[24] Borrelli MR, Shen AH, Lee GK, Momeni A, Longaker MT, Wan DC. Radiation-induced skin fibrosis: pathogenesis, current treatment options, and emerging therapeutics. Ann Plast Surg. 2019;83(4S)(suppl 1):S59-S64. doi:10.1097/SAP.0000000000002098 31513068 PMC6746243

[25] Pappo AS, Armstrong GT, Liu W, . Melanoma as a subsequent neoplasm in adult survivors of childhood cancer: a report from the Childhood Cancer Survivor Study. Pediatr Blood Cancer. 2013;60(3):461-466. doi:10.1002/pbc.24266 22887858 PMC3538914

[26] Mermershtain W, Cohen AD, Koretz M, Cohen Y. Cutaneous angiosarcoma of breast after lumpectomy, axillary lymph node dissection, and radiotherapy for primary breast carcinoma: case report and review of the literature. Am J Clin Oncol. 2002;25(6):597-598. doi:10.1097/00000421-200212000-00014 12478007

[27] Darby S, McGale P, Correa C, ; Early Breast Cancer Trialists’ Collaborative Group (EBCTCG). Effect of radiotherapy after breast-conserving surgery on 10-year recurrence and 15-year breast cancer death: meta-analysis of individual patient data for 10,801 women in 17 randomised trials. Lancet. 2011;378(9804):1707-1716. doi:10.1016/S0140-6736(11)61629-2 22019144 PMC3254252

[28] Geller AC, Keske RR, Haneuse S, . Skin cancer early detection practices among adult survivors of childhood cancer treated with radiation. J Invest Dermatol. 2019;139(9):1898-1905.e2. doi:10.1016/j.jid.2019.02.033 30959042 PMC6708770

[29] Watts CG, McLoughlin K, Goumas C, . Association between melanoma detected during routine skin checks and mortality. JAMA Dermatol. 2021;157(12):1425-1436. doi:10.1001/jamadermatol.2021.3884 34730781 PMC8567188

[30] Aggarwal P, Knabel P, Fleischer AB Jr. United States burden of melanoma and non-melanoma skin cancer from 1990 to 2019. J Am Acad Dermatol. 2021;85(2):388-395. doi:10.1016/j.jaad.2021.03.109 33852922

[31] Jagsi R, Abrahamse P, Hawley ST, Graff JJ, Hamilton AS, Katz SJ. Underascertainment of radiotherapy receipt in Surveillance, Epidemiology, and End Results registry data. Cancer. 2012;118(2):333-341. doi:10.1002/cncr.26295 21717446 PMC3224683

