# Supplementary material for: Incidence of Nonkeratinocyte Skin Cancer After Breast Cancer Radiation Therapy
Source: JAMA Netw Open. 2024 Mar 8;7(3):e241632. doi: 10.1001/jamanetworkopen.2024.1632 (PMC10924238; doi:10.1001/jamanetworkopen.2024.1632)
Supplement: Supplement. — Data Sharing Statement [file jamanetwopen-e241632-s001.pdf]

Data Sharing Statement

Rezaei. Incidence of Nonkeratinocyte Skin Cancer After Breast Cancer Radiation Therapy. *JAMA Netw Open*. Published March 08, 2024. doi:10.1001/jamanetworkopen.2024.1632

Data
Data available: No

Additional Information
Explanation for why data not available: This article uses a publicly available data source, the CDC's SEER registry, for the analysis
